# Saccharated Calomel

**Published:** 1874-08

**Authors:** W. H. Saxon

**Affiliations:** Dirt Town, Georgia


### SACCHARATED CALOMEL.

By W. H. SAXON, M.D., Dirt Town, Georgia.

*Editor Atlanta Medical and Surgical Journal:*

As there seems to be some diversity of opinion among the profession as to the origin of saccharated calomel, I write to state that, by reference to the valuable treatise on children, by Dr. Dewes, pages 274 and 402, you will find the mode of administration in minute doses, by no means a new one, in jaundice and cholera infantum in children, and in cholera in adults. The following was his formula in 1795:

℞.—Calom. ppt. . . . . . . . gr. iij.
Sacch. alb. . . . . . . . . gr. vj.
M. div. in par., No. xij.

One powder to be thrown into the patient's mouth dry, every two or three hours, until biliary evacuations follow.

